# An Experimental Study in Wild Wood Mice Testing Elemental and Isotope Analysis in Faeces to Determine Variations in Food Intake Amount

**DOI:** 10.3390/ani13071176

**Published:** 2023-03-28

**Authors:** Álvaro Navarro-Castilla, M. Carmen Hernández, Isabel Barja

**Affiliations:** 1 Eco- and Ethophysiology Lab, Departamento de Biología, Universidad Autónoma de Madrid, 28049 Madrid, Spain; 2 Centro de Investigación en Biodiversidad y Cambio Global (CIBC-UAM), Universidad Autónoma de Madrid, C. Darwin 2, 28049 Madrid, Spain

**Keywords:** *Apodemus sylvaticus*, diet, feeding behaviour, rodents, stable isotopes, trophic ecology

## Abstract

**Simple Summary:**

Elemental and stable isotope analyses are useful and common methods for wildlife diet studies, e.g., for characterizing diets and trophic relationships. However, little is known about the potential applicability of these techniques to address other aspects of feeding ecology. Here, we evaluated whether faecal elemental (carbon and nitrogen) and/or isotopic values (δ^13^C, δ^15^N) can determine variations in the amount of food intake. Overall, elemental analyses and δ^15^N values failed in reporting significant differences, but preliminary outcomes support the potential use of faecal δ^13^C values as an indicator to detect short-term slight food intake changes. The results of this work provide, for the first-time, reference data for interpreting faecal elemental and isotopic patterns in free-ranging wood mice (*Apodemus sylvaticus*), as well as new insights into the additional applicability of isotopic analysis in feeding ecology studies.

**Abstract:**

The analysis of carbon and nitrogen elemental (C, N) and isotopic compositions (δ^13^C, δ^15^N) in faeces are considered reliable methodologies for the study of diet in wildlife. Here, we tested the suitability of these techniques to detect variations in the amount of food intake. We captured wild wood mice (*Apodemus sylvaticus)* with Sherman live traps where bait access was initially free, and later it was experimentally limited inside by four different devices to cause intended variations in the amount ingested. The total C and N (%) and stable δ^13^C and δ^15^N isotopic values were determined for the bait and in mice faecal samples. Faecal values were lower than bait ones except for N, likely due to animal matter ingested before capture. No significant differences in total C, N and δ^13^C were found due to individual traits. However, breeding males showed higher δ^15^N values than breeding females, probably due to differences in energy and protein demands between both sexes during the breeding season. Only δ^13^C detected food intake variations (≥2 g). Despite further research being needed, these results initially support the potential of δ^13^C to provide information on the amount ingested, thus being useful to complement trophic ecology studies.

## 1. Introduction

Nutrition is a key regulator of animals’ growth, survival and reproductive success; therefore, studies on nutritional physiology and ecology of wildlife are pivotal [1,2,3]. In nature, the nutritional status of individuals is conditioned by the quality and/or quantity of available food resources. Individuals living in the same habitat have, a priori, access to similar resources, but foraging may also involve costs, becoming especially challenging when the access to food is particularly difficult, restricted or entails high energetic expenditures. Hence, the degree of exploitation may also depend on each individual’s needs, but also on their efforts and experience to overcome any limiting barriers [4,5,6]. Furthermore, environmental factors (e.g., competition, predation risk and weather conditions) and individual traits can also strongly impact body condition and energetic demands of individuals, as well as feeding behaviour [7,8,9,10,11,12]. Thus, studies on feeding ecology are paramount for assessing the status of wild animal populations as well as to understand the wildlife relationship with the habitat and environmental changes [3]. However, addressing diet composition and food intake in free-ranging animals turns challenging due to several constraints [13], e.g., the costly and time-consuming drawbacks of direct observations of feeding habits in the field. Similarly, the study of gastrointestinal contents also comprises logistical challenges plus ethical issues as it involves invasive procedures or the sacrifice of animals. Alternatively, faecal analysis of food remains represents a useful non-invasive option for studying the diet [13,14,15,16,17]. Furthermore, advances in molecular techniques have increased the possibilities of faecal sampling as a powerful research tool for monitoring feeding habits, but also for targeting other aspects of wildlife biology [18,19,20,21,22,23,24].

Today, elemental and stable isotopes analyses are well-stablished tools to assess wild animals’ diet and foraging ecology in a wide range of animal tissues as well as in urine and excreta samples [25,26,27,28,29,30,31,32,33]. Overall, the elemental analysis of faecal material allows us to gather valuable information as the carbon and nitrogen percentages may indicate the nutritional quality of the diet, i.e., the availability of resources and/or how they are being exploited [34,35]. Particularly, faecal nitrogen has been considered as a reliable indicator of diet quality [36,37,38] as it is considered a limiting resource [39]. Thus, elemental analysis has been extensively used to understand the nutritional ecology of wildlife species, including small mammals [40,41,42,43]. Similarly, the use of carbon and nitrogen stable isotopes in trophic ecology is widespread as ^13^C/^12^C and ^15^N/^14^N isotopic ratios archived in animal tissues or excreta are directly related to those in their diet [44,45,46]. Carbon isotope ratio mainly determines differences between C3 and C4 plant consumption, as they differ in their photosynthesis pathway (e.g., CO_2_ fixation enzymes involved and first stable fixation products) leading to different ^13^C/^12^C (i.e., δ^13^C) ranges (C3 plants: −20 to −37‰, C4 plants: −9 to −17‰). Conversely, nitrogen isotopes are related to trophic behaviour and conditioning environmental factors [44,47]. Faecal isotopic compositions provide insight about an animal’s diet during a brief period, presumably allowing the examination of short-term dietary changes [48,49,50]. However, despite both elemental and isotopic methodologies having been proven to be useful tools for characterizing diets and trophic relationships, the potential applicability of these analytical techniques to address other aspects of feeding ecology, e.g., detecting variations in the amount of food intake in wildlife, is poorly understood.

Our main goal was to test whether faecal elemental and isotopic values can determine changes in the amount of food intake. For this, we captured wild wood mice (*Apodemus sylvaticus*) using toasted corn as bait, but the access was experimentally manipulated to achieve differences in the amount ingested. The wood mouse is one of the most common and widespread rodent species; however, little is known about its isotopic composition [51,52]. Furthermore, we consider it important to highlight that, to the best of our knowledge, there are a lack of data on elemental carbon and nitrogen content for this species in the literature, and no other works have previously analysed the elemental and isotopic signatures in faecal samples. Hence, we analysed the diet/faeces trophic discrimination factor for this species and faecal C and N content (%), as well as δ^13^C and δ^15^N composition were studied in relation to individual traits (sex, age and breeding condition). Our two primary hypotheses and predictions were (1) Considering the differences in the amount of food intake experimentally caused between individuals, we expect mice to show variation in the faecal elemental and isotopic values; (2) In the same way, as individuals may exploit resources differently and/or have different energetic demands, intraspecific differences (due to individual traits) are also anticipated to be a source of variation for these outputs. The obtained data are compared and discussed with results from other rodent species.

## 2. Materials and Methods

### 2.1. Field Sampling

The study was conducted in the “Monte de Valdelatas” Mediterranean forest (Madrid, Spain, 40°32′ N 3°41′ W). It is located at an altitude of 650 m a.s.l. and the vegetation is mainly characterised by holm oak forests (*Quercus ilex ballota*) and shrub communities, including species such as gum rock roses (*Cistus ladanifer*), umbel-flowered sun roses (*Halimium umbellatum*) and thyme (*Thymus zygis*). 

Free-ranging wood mice were live-captured using Sherman^®^ traps (H.B. Sherman Traps, Tallahassee, FL, USA) baited with 5 g of toasted corn (*Zea mays*). Trapping was conducted during 15 consecutive days, being simultaneously replicated in 4 different sites established along the study area and separated by 35 m. Each sampling site was 0.59 h in area and consisted of 20 traps set up shaping a 4 × 5 grid and spaced 7 m apart [5,6]. Trap effort was 1200 trap-nights (4 grids × 20 traps × 15 nights). To minimize mice exposure to the potential adverse climatic conditions during trap confinement, we positioned traps under the shrub cover. Furthermore, to avoid capturing non-target species as well as longer confinement periods, traps were activated daily at dusk and checked the next day early in the morning.

Captured individuals were identified at the species level by morphological criteria [53]. Sex and breeding condition were assessed by visually inspecting genitals (males: enlarged scrotum, females: perforated vagina, prominent nipples, signs of pregnancy or lactation; [53]) and age class (juvenile, subadult or adult) was estimated based on body weight (measured using a PESNET 100 g spring-scale; [54]). Mice were marked with harmless waterproof paints (Marking stick DFV, www.divasa-farmavic.com, accessed on 16 September 2022) in different body parts to allow recognition of recaptures, and then individuals were released at the point of capture. 

### 2.2. Food Access Limitation and Food Intake Evaluation

All traps (80) were firstly treated as controls (free access to the 5 g of toasted corn used as bait) during 3 consecutive nights. Following this, access to bait was experimentally manipulated by providing the 5 g of bait inside a different device every 3 nights (we used 4 devices made with straw, metal wire or plastic bottles, see specific details in [5,6]) which conditioned food access and consequently food intake amount. Food intake was estimated as the difference between the 5 g of bait provided and the unconsumed amount of toasted corn left in the trap by each captured mouse, weighed with an electronic balance (C-3000/0.01 g CS, COBOS; precision 0.01 g). 

### 2.3. Faeces Collection and Elemental and Isotope Analyses

Faecal samples consisted of a collection of faecal pellets non-invasively obtained every morning from each trap. Only fresh faecal pellets were selected, avoiding urine contaminated faeces to prevent any result bias. Faecal samples were stored in Eppendorf tubes inside a portable cooler with ice (4 °C) and later in the laboratory frozen at −20 °C.

Before being processed, faeces were oven-dried at 90 °C for 4 h to remove water content. Later, dried faecal samples were milled into a homogenous powder and stored in Eppendorf tubes. Faecal samples, together with a milled mix sample of the toasted corn used as bait, were sent to the Interdepartmental Research Service (SIdI) of the Autonomous University of Madrid for performing the analyses for elemental concentrations and ^13^C/^12^C and ^15^N/^14^N ratios. The microanalysis of total Carbon (C) and Nitrogen (N) content, and ^13^C/^12^C and ^15^N/^14^N ratios were analysed using a continuous flow Thermo Delta V Advantage isotopic ratio mass spectrometer (IRMS) coupled to an Elemental analyzer Thermo EA 1112 HT. Analyses were calibrated with internal standards, determining an analytical accuracy (based on repeated measurements for replicate samples) of ±0.42% C and ±0.22% N for the elemental analysis, and ±0.20‰ and ±0.18‰ for δ^13^C and δ^15^N, respectively. Total C and N are expressed in percentage (%) and isotopic signatures are shown in conventional delta notation (δ) per mil (parts per thousand, ‰) with respect to the Vienna Pee Dee Belemnite (VPDB) standard for C (δ^13^C_VPDB_) and the atmospheric air standard (AIR) for N (δ^15^N_AIR_). The delta notation expresses the variation of the ratio of the heavy to light stable isotope in the sample (i.e., δ^13^C = ^13^C/^12^C and δ^15^N = ^15^N/^14^N) relative to the same isotopic ratio of the corresponding standards (VPDB and AIR, respectively).

### 2.4. Statistical Analysis

Since the main goal of this research was to evaluate whether changes in the amount of food intake can be detected by elemental and isotopic analyses in faeces, we previously analysed food intake variation and established different groups based on mean and range values. Subsequently, differences between groups were analysed for statistical significance though a General Lineal Model (GLM) and Tukey’s honestly significant difference tests (Tukey’s HSD). To control for the possible influence of individual traits, the interactions food group * sex, food group * breeding and food group * age, as well as the individual and recapture, were also included as fixed factors in the GLM.

The elemental and isotopic values of the bait (toasted corn, N = 5) and mice faeces (N = 113) were analysed for statistical differences through independent Student’s *t*-tests. Diet/faeces trophic discrimination factors (TFD) were calculated as the difference between the bait and faecal data, i.e., the mean values of the toasted corn were subtracted to the mean faecal values [30].

The statistical models applied for analysing elemental C and N in faeces did not meet the assumptions (e.g., distribution and variance of model residuals) required to report valid results. Consequently, and owing to the size of our dependent variables, we draw conclusions by using Student’s *t*-test (for sex and breeding condition) or ANOVA (for food groups and age) tests to compare the potentially existing differences in C and N values between levels of the same factor.

The variation in the isotopic response variables (i.e., δ^13^C and δ^15^N) was analysed by performing generalized linear mixed models (GLMM, fitting normal distribution and identity-link function), including as fixed factors the pure effects of food group (<1 g; 1–<2 g; 2–<3 g; 3–<4 g; 4–5 g, see details below), sex (male/female), breeding condition (non-breeder/breeder) and age (juvenile/subadult/adult). Since metabolism differences due to the combination of sex and breeding condition have been reported in different species, this factor interaction (indicated by an asterisk “*”) was also included in the statistical models to control for its possible interactive effect. No other interaction between individual factors was included due to the lack of biological relevance (age * breeding) and/or because data distribution did not allow appropriate analysis (age * sex). Furthermore, as some mice were recaptured, the recapture * individual interaction was included as a random factor to control for any possible inter-individual variation. Assumptions of normal distribution and homogeneity of variances were checked in the residuals of the GLMMs carried out. 

We used SPSS 22.0 for Windows (SPSS Inc., Chicago, IL, USA) software to carry out the statistical analysis. Results were considered significant at α < 0.05 and data are shown as mean ± standard error (SE).

## 3. Results

Based on the significant variation found in the amount of food intake among captured individuals, the following five different groups of food intake were established: (1) 0–0.97 g (mean ± SE: 0.54 ± 0.09; n = 13), (2) 1.18–1.98 g (mean ± SE: 1.64 ± 0.06; n = 20), (3) 2.03–2.94 g (mean ± SE: 2.54 ± 0.05; n = 28), (4) 3.06–3.92 g (mean ± SE: 3.50 ± 0.06; n = 26) and (5) 4.02–5.00 g (mean ± SE: 4.64 ± 0.07; n = 26) (*F*_4,113_ = 90.052, *p* < 0.0001; Tukey’s HSD, *p* < 0.0001 for all cases). No statistically significant differences were detected in food intake within each food group due to individual traits, i.e., food group * sex (*F*_4,113_ = 1.250, *p* = 0.304), food group * breeding (*F*_5,113_ = 1.791, *p* = 0.134) and food group * age (*F*_8,113_ = 0.633, *p* = 0.745). Furthermore, neither the individual (*F*_42,113_ = 1.052, *p* = 0.432) nor the recapture (*F*_1,113_ = 0.061, *p* = 0.807) resulted as significant factors explaining the variation in food intake. 

The toasted corn provided as bait in this study had the following elemental (C = 47.30%; N = 1.10%) and isotopic (δ^13^C = −13.90‰, range: −13.65 to −14.19; δ^15^N = 5.40‰, range: 5.29–5.56) composition. Elemental and isotopic values were analysed in a total of 113 faecal samples from 43 different wood mice. Overall, faecal values were lower than those obtained in the bait, except for total N, and this pattern was observed in all faecal samples independently of the sex, breeding condition and age of individuals (Table 1). Compared to bait values, this resulted in a 3.32% reduction for C and a 3.73% N increase in faecal samples. Mean δ^13^C and δ^15^N TDFs were −9.01‰ and −1.51‰, respectively. Nevertheless, these variations between corn and faecal values were only statistically significant for N (*p* < 0.0001) and δ^13^C (*p* < 0.0001), but not for C (*p* = 0.121) and δ^15^N (*p* = 0.110). 

No significant differences were found in total C and N due to sex, breeding condition or age of individuals (see mean values in Table 1, *p* > 0.05 in all cases). In addition, differences in the amount of food intake did not lead to significant variation of total C and N values (Table 2). Conversely, the GLMM analysing δ^13^C data revealed that food group was the only factor significantly explaining the variation found in δ^13^C values (Table 3).

**Figure 1:**
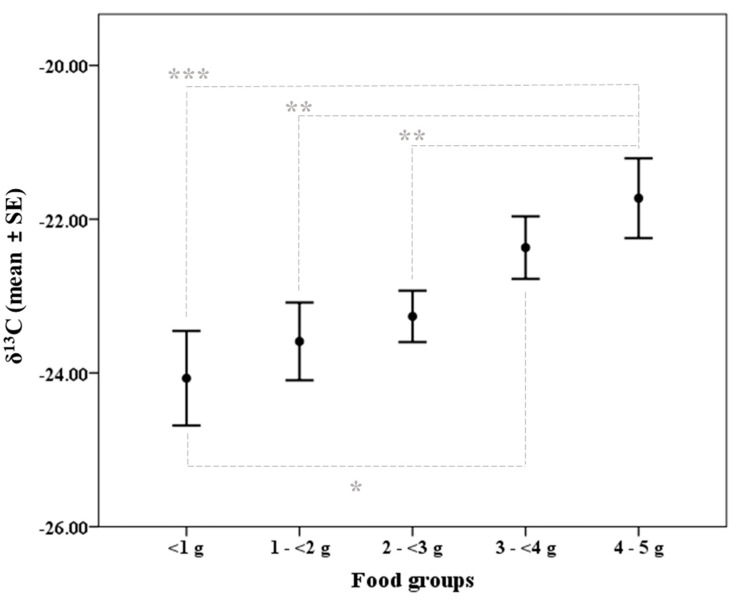
δ^13^C values in relation to food groups. Grey broken lines and associated asterisks indicate significant differences between connected groups (* *p* < 0.05, ** *p* < 0.01, *** *p* < 0.001).

**Figure 2:**
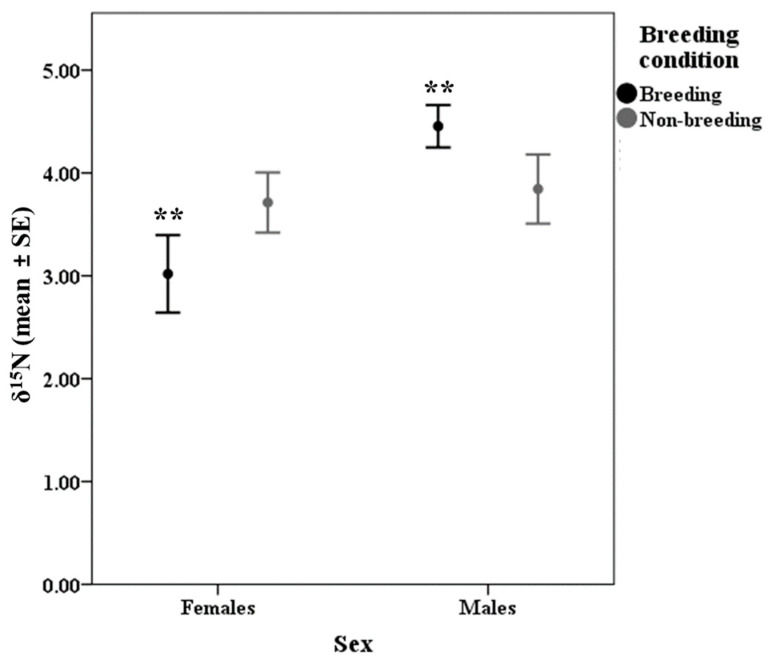
δ^15^N values due to sex and breeding condition of individuals. Asterisks indicate significant differences (** *p* < 0.01).

Results showed that δ^13^C values could determine food intake differences (Figure 1), concretely significant differences in δ^13^C were found between group 1 (<1 g) compared to groups 4 (3–<4 g; *p* = 0.031) and 5 (4–5 g; *p* < 0.0001). Furthermore, food group 5 (4–5 g) also presented significant differences in δ^13^C values with food groups 2 (1–<2 g; *p* = 0.001) and 3 (2–<3 g; *p* = 0.006). Regarding δ^15^N results (Table 3), values found for each food group were as follows: <1 g = 3.81 ± 0.39, 1–<2 g = 3.18 ± 0.29, 2–<3 g = 4.06 ± 0.35, 3–<4 g = 4.01 ± 0.33, and 4–5 g = 3.88 ± 0.15. However, the differences were not statistically significant. Sex appeared in the model as a significant factor influencing δ^15^N variation. However, the significant interaction between sex * breeding condition showed that differences in δ^15^N values between males and females were only significant for breeding individuals (*p* = 0.002; Figure 2). 

## 4. Discussion

In this study, we used faecal samples non-invasively collected to test whether elemental and isotopic values observed in faeces are representative of the amount of food intake by wild wood mice. The toasted corn used as bait showed an elemental composition (percentages of carbon and nitrogen) like the available data reported for this food item and the corn plant [55,56,57,58]. Similarly, the isotopic composition of the bait, i.e., δ^13^C and δ^15^N, also matched values described in the literature for the plant and derived products, including this food type [30,56,59]. 

Regarding the elemental and isotopic composition registered in wood mouse faeces, in comparison with corn data, we detected a significant N enrichment. The animal’s metabolism and assimilation to meet nutritional requirements may explain these variations between values observed for corn and faeces. Furthermore, considering that the gastrointestinal transit time of ingesta lasts several hours in mice [60,61,62], we cannot exclude that the elemental and isotopic information registered in faeces could likely also be influenced by the diet consumed before the experiment. For example, the significant N enrichment noted could be due to animal material previously ingested, since the wood mouse also feeds on arthropods and other invertebrates [63,64]. Similarly, the significant lower δ^13^C values found in faeces would be well-supported by the natural diet of the wood mouse, which is mainly comprised of vegetal matter (i.e., seeds, fruits, roots, and other plant parts [63]) resulting from C3 plants (see above description of the vegetation in the study area), where carbon isotope composition ranges from −20 to −37‰ [65]. This depletion in faecal δ^13^C values as compared with the diet has been also reported by other works carried out with several rodents, including the yellow-necked mouse *Apodemus flavicollis* [49,66]. Furthermore, both elemental and isotopic values found for the wood mouse in this study are consistent with the ranges found for other rodent species both in faeces [34,49,66,67,68,69,70] and hair samples [67,71,72,73,74,75,76,77,78]. Furthermore, the faecal isotopic composition of wood mouse faeces found also falls within values reported in the few available scientific works addressing hair and blood isotopic composition in the wood mouse [51,52]. 

Contrary to our predictions, no significant differences in elemental and isotopic values were found due to the pure effect of age, sex and breeding condition. We have not found other rodent studies specifically analysing in faecal samples the elemental carbon and nitrogen as a function of individual characteristics; hence, we are limited in making appropriate comparisons and interpreting how different factors may have influenced the elemental composition found in the wood mouse. Palo and Olsson [79] found that in the bank vole (*Myodes glareolus*), concentrations of nitrogen as well as the ratio of nitrogen to carbon in stomach content were not different between the sexes. In addition, our results would be also supported by a rodent study (including *Apodemus* spp.) in which authors found no gender differences in the concentration of various chemical elements in muscle and bone samples [80]. Likewise, no differences in isotopic values depending on age, sex or breeding condition have been found in several rodent species, including the wood mouse [51,70,74,75,81,82,83,84]. Overall, the non-variation in elemental and isotopic values due to individual traits could be attributed to the observed lack of individual differences in food intake inside traps. Furthermore, since differences in the wood mouse diet due to age, gender and sexual activity have been reported to occur infrequently [63,85], arguably, available resources in the study area may be being equally used [84]. This will be also supported considering that half-lives of isotopic values have been reported to be from several days to months (depending on the tissue, i.e., hair, blood, organs…) in rodents and other mammal species [44,86,87,88]. In fact, this could also back that significant higher δ^15^N values found in breeding males compared to breeding females are due to a synergy between the natural diet and differential requirements, since (i) occasionally, male stomachs have been shown to contain more animal food than female ones [63,89,90] and (ii) protein requirements of breeding females are highly increased due to the physiological costs associated with both gestation and lactation [91,92]. Thus, we speculate that the increased exploitation of dietary nitrogen for tissue synthesis and deposition by breeding females resulted in a reduction in faecal excretion and this would explain the depleted δ^15^N values [93]. 

Our experimental set-up successfully led to different food intake groups; however, this just allowed us to partially confirm our predictions regarding the potential of elemental and isotopic analyses to determine variations in the amount of food intake. Based on our results, faecal carbon and nitrogen total concentrations do not seem to be sufficiently impacted by small variations in food intake. Thus, although elemental analysis has proven to be a useful tool to assess diet characteristics, it would not be suitable for detecting small-scaled changes in the amount of food intake. Contrarywise, δ^13^C values were able to detect food intake differences starting at 2 g, but the sensitivity seems to be conditioned by the amount ingested since this 2 g accuracy was only achieved when individuals ingested 4–5 g; while, if individuals consumed 3–<4 g, food intake differences should be at least 3 g to be significantly reflected in δ^13^C values. Conversely, δ^15^N values observed in faeces did not determine food intake variations at any level. A similar result was found in African spiny mice (*Acomys spinosissimus*) where individuals subjected to a 10% food-restricted diet showed no significant differences in δ^15^N in blood and liver samples, while ^13^C values were depleted [94]. As discussed by these authors, it is possible that the low nitrogen content of the bait and/or food intake differences were not relevant enough to cause alterations in δ^15^N values. Moreover, considering the nitrogen half-life in animal tissues, likely the plant protein-based bait provided was not enough to cause a substantial shift in δ^15^N turnover and reflect, through faecal isotopic values, the changes in the amount ingested [95]. Thus, further work in faecal δ^15^N is still needed to be considered a reliable proxy for determining food intake variations in wildlife.

## 5. Conclusions

In summary, as far as we know, this is the third study reporting isotopic values in the wood mouse [51,52], but the first one performed on faeces and including elemental data. Thus, this work represents a novel and valuable contribution to the knowledge about the faecal carbon and nitrogen values in this species. It also helps to improve the understanding of diet/faeces trophic discrimination factors. We can conclude that the results of this work provide, for the first time, reference data for interpreting faecal elemental and isotopic patterns in free-ranging wood mice, as well as new insights into the additional applicability of isotopic analysis in feeding ecology studies. We argue that these preliminary outcomes support the potential use of faecal δ^13^C values as an indicator to detect short-term slight food intake changes. However, further investigation (e.g., testing different food items of a species typical diet in the nature, as well as mixtures of these and amount combinations) is still required to fully exploit this methodology in wildlife trophic studies.

## Figures and Tables

**Table 1:** Elemental (C and N in %) and isotopic (δ^13^C and δ^15^N in ‰) values obtained in wood mouse faecal samples.

		C		N		δ^13^C		δ^15^N	
		Mean	SE	Mean	SE	Mean	SE	Mean	SE
Pooled data		43.98	0.34	4.83	0.08	−22.91	0.22	3.89	0.15
Range (min-max)		(27.80–50.30)		(3.10–9.00)		(−27.60–−17.30)		(0.20–8.30)	
Sex	male	43.45	0.49	4.84	0.15	−22.92	0.29	4.18	0.19
	female	44.37	0.47	4.83	0.10	−22.90	0.34	3.48	0.23
Breeding condition	breeder	43.72	0.52	4.85	0.13	−22.47	0.31	4.02	0.20
	non-breeder	44.21	0.45	4.81	0.11	−23.28	0.29	3.78	0.22
Age	juveniles	44.12	1.58	4.58	0.25	−23.13	0.90	3.77	0.63
	subadults	44.09	0.52	4.88	0.15	−23.53	0.36	3.63	0.26
	adults	43.97	0.48	4.84	0.11	−22.48	0.28	4.09	0.20

**Table 2:** Faecal C and N values in relation to food intake.

		C (%)			N (%)		
		Mean	SE	Statistic	Mean	SE	Statistic
Food groups	<1 g	44.08	1.02	ANOVA *F*_4,113_ = 1.284, *p* = 0.281	4.90	0.24	ANOVA *F*_4,113_ = 0.545, *p* = 0.703
	1–<2 g	42.88	0.94		4.85	0.20	
	2–<3 g	44.53	0.51		4.77	0.22	
	3–<4 g	43.31	0.92		4.65	0.14	
	4–5 g	44.94	0.55		5.01	0.16	

**Table 3:** Results from the GLMMs analysing the variation in isotopic δ^13^C and δ^15^N values in relation to food groups and individual factors.

	δ^13^C			δ^15^N		
Factors	df	*F*	*p*	df	*F*	*p*
Corrected model	9	2.537	0.011	9	2.293	0.022
Sex	1	2.303	0.132	1	6.110	0.015
Breeding condition	1	0.018	0.892	1	0.054	0.817
Age	2	1.051	0.353	2	0.166	0.848
Food group	4	4.468	0.002	4	2.241	0.070
Sex * Breeding condition	1	0.038	0.846	1	4.582	0.035
Error	102			102		
Recapture * Individual ª						

ª Random factors. δ^13^C (Estimate: 1.243, SE: 0.763, Z-test: 1.629, *p*: 0.103). δ^15^N (Estimate: 1.615, SE: 0.611, Z-test: 2.643, *p*: 0.008).

## Data Availability

Data is unavailable due to privacy.
